# Bactericidal Effects of Pulsed-Light Treatment Against *Burkholderia gladioli* pv. *cocovenenans* in *Auricularia*: Mechanisms and Influences

**DOI:** 10.3390/foods14132246

**Published:** 2025-06-25

**Authors:** Chen Niu, Jin Hao, Zeyu Hu, Yuchen Song, Yilin Ren, Yuanchun Wu, Jing Yang, Zihan Song, Yahong Yuan, Tianli Yue

**Affiliations:** 1 College of Food Science and Technology, Northwest University, Xi’an 710069, China; 2 Laboratory of Nutritional and Healthy Food-Individuation Manufacturing Engineering, Xi’an 710069, China; 3 Research Center of Food Safety Risk Assessment and Control, Xi’an 710069, China; 4 Institute of Vegetables and Flowers, Chinese Academy of Agricultural Sciences, Zhongguancun South Street, Haidian District, Beijing 100081, China; songzihan@caas.cn

**Keywords:** pulsed light, inactivation, *Burkholderia gladioli* pv. *cocovenenans*, *Auricularia*

## Abstract

*Auricularia* (wood ear fungus) is susceptible to *Burkholderia gladioli* pv. *cocovenenans* (BGC) and causes food poisoning. This study investigated pulsed-light (PL) inactivation of BGC on *Auricularia*. The evaluation of PL parameters indicated that lower initial biomass, shorter distance, and more fluences were more effective in inactivating BGC. This study achieved 5~8 Log reductions in BGC in phosphate-buffered saline and ~4 Log reductions in *Auricularia auricula* and *Auricularia cornea* var. Li, and the survivor curves fit the Weibull model well with R^2^ values of 0.943~0.987 and RMSE values of 0.308~0.430 in all three substrates. PL caused cell membrane damage, leading to DNA, protein, and ATP leakage and increased ROS production. PL caused an alteration in color (ΔE 3.01~7.67) but not springiness and cohesiveness in the texture of *Auricularia* at 11.80~35.40 J/cm^2^. In all, PL is effective in inactivating BGC on the surface of *Auricularia* at 35.40 J/cm^2^ and can be taken as a good controlling measure.

## 1. Introduction

*Auricularia* (wood ear fungus) is the third largest edible mushroom in China and one of the four largest edible mushrooms in the world [1]. With the promotion of the concept of big food, *Auricularia* is widely loved by consumers because of its beneficial nutrients and active substances. It has been shown that it has anti-dementia effects in the treatment of ischemic stroke with metabolic syndrome (MetS) [2]. The ferment of *Auricularia* has a preventive effect on liver and stomach injury caused by alcohol poisoning [3]. And the polysaccharides of *Auricularia* can alleviate many chronic diseases by improving intestinal flora, regulating inflammatory response, and alleviating oxidative stress damage and lipid metabolism [4,5].

However, *Auricularia* is infested with the bacterium *Burkholderia gladioli* pv. *cocovenenans* (BGC), which grows in abundance during the rehydration process of its dried products, and BGC is the only pathogenic variant of *Burkholderia* that can cause food poisoning in humans [6]. It is widely distributed in nature and is also found in human foods such as fermented cereals and fresh *Tremella* and *Auricularia*. It produces bongkrekic acid and toxoflavin and is responsible for several severe cases of food poisoning in Asia and Africa [7]. Bongkrekic acid is a mitochondrial adenine nucleotide translocator (ANT)-specific binding toxin that inhibits the respiratory chain and causes liver damage and even death [8,9,10]. It is colorless, tasteless, heat-stable, and usually not detectable in food. Toxoflavin is a water-soluble yellow toxin that has antibiotic activity against a variety of bacteria and fungi. It acts as an electron carrier between oxygen and NADH, during which process hydrogen peroxide is produced, thereby increasing the level of reactive oxygen species and destroying cell structure, an initiation of cytotoxicity [11]. Both toxins have made BGC infamous as one of the most lethal microbes, endangering food safety and consumer health.

Although *Auricularia* shows great potential in the fields of nutrition and medicine, its production and consumption face serious challenges due to the presence of BGC. In order to protect the health of consumers, effective prevention and control measures must be taken to prevent BGC from impacting the safety of *Auricularia*.

Pulsed light is an emerging non-thermal processing technology and is accepted by the FDA for food surface sterilization [12]. The structure of pulsed-light (PL) equipment includes the power supply, processing chamber, and cooling system, as well as the core component, the xenon lamp. During operation, a pulsed current excites the xenon lamp to produce pulsed light with a broadband spectrum wavelength between 200 and 1100 nm [13,14,15]. The bactericidal mechanism of pulsed light is ascribed to the effect of the ultraviolet spectral range on biological DNA molecules, usually DNA breakage and chemical modifications [16,17]. In general, photothermal, photochemical, and photophysical effects contribute to the inactivation of biological cells and damage on their structures [14]. Pulsed light is a non-thermal technology that is residue-free, causes minimal changes in quality [18], and is suitable for the surface disinfection of solid or transparent liquid food [19]. The duration of second-level rapid processing stands out from that of other novel non-thermal technologies such as high-pressure processing and cold plasma [20].

To date, there are limited reports on the effective control of BGC, especially non-thermal processing technologies that exert minimal deterioration on *Auricularia* food quality. Therefore, the aim of this study is to evaluate the efficacy of PL on the inactivation of BGC on *Auricularia*. The inactivation effect of PL on BGC and the influence of PL parameters on the inactivation were evaluated. The bactericidal mechanism was further investigated. In addition, the changes in the color and texture of *Auricularia* after PL treatment were assessed.

## 2. Materials and Methods

### 2.1. Microorganisms and Equipment

*Burkholderia gladioli* pv. *cocovenanans* NC18 (NCBI accession No. OP984861.1) isolated from *Auricularia auricula* in the planting base of ZhaShui County, Shang Luo, Shaanxi province (109.14° E, 33.69° N) was used throughout this work [21].

PL equipment (FD-2000, Pulsed Light Power BV, Eindhoven, The Netherlands) with a 25 cm length xenon lamp was used in this work. The device produces high-intensity pulses of light, has a rated voltage of 1320 V, and can release 1850 J of energy per flash. According to the specifications, the light fluence generated by each flash at 10 cm, 15 cm, and 30 cm is 1.18 J/cm^2^/flash, 0.79 J/cm^2^/flash, and 0.59 J/cm^2^/flash, respectively.

### 2.2. Inactivation Studies

BGC was cultured in Potato Dextrose (potato extract 12 g/L and dextrose 20 g/L) broth overnight. Then, the cells were washed in PBS and collected by centrifugation at 5000× *g*, 10 min, and 4 °C. The cells were adjusted to proper biomass for subsequent use.

The effects of initial biomass (9 Log CFU/mL, 8 Log CFU/mL, and 7 Log CFU/mL), distance from lamp (10 cm, 15 cm, and 20 cm), and number of flashes (1~5 times corresponding to 1.18~5.90 J/cm^2^) on the inactivation of BGC strains were considered based on equipment settings and prior experiments. A bacterial suspension (6 mL) was placed on a Petri dish (60 mm) for PL treatment.

Dry *Auricularia auricula* (AA) and *Auricularia cornea* var. Li. (AC) were sterilized in an autoclaver at 121 °C for 20 min, and ultrapure water was added for rehydration for 2 h. BGC bacterial suspensions (200 μL) were added dropwise onto the pieces of AA and AC. The distance to the lamp was kept at 10 cm, and the fluences were 5.9, 11.8, 17.7, 23.6, 29.5, and 35.4 J/cm^2^. The bacteria were recovered for colony enumeration. The plate counting method was employed to evaluate the inactivation effect of PL on BGC. In total, 100 μL of treated samples were plated on Potato Dextrose Agar for 48 h at 37 °C. The colony-forming units were recorded. Untreated bacterial suspensions were taken as a blank control. The experiment was conducted in three replicates.

The inactivation of BGC on PBS, AA, and AC was fitted using log-linear and Weibull models, and the best-fit model was chosen to simulate the bacterial inactivation trend. The Geeraerd and Van Impe Inactivation Model Fitting Tool (GInaFiT version 1.8) plug-in was loaded into Microsoft Excel for model fitting [22].

### 2.3. Scanning Electron Microscopy (SEM) and Transmission Electron Microscopy (TEM)

BGC bacterial cells dispersed in 0.01 M PBS (approximately 108 CFU/mL) were treated with PL at 2.36 and 5.90 J/cm^2^. Morphological changes in bacterial cells were observed by SEM (Sigma300, Carl Zeiss AG, Baden-Württemberg, Germany). After PL treatment and centrifugation (5000× *g*, 10 min, and 4 °C), the suspensions were fixed with 2.5% glutaraldehyde at 4 °C for 12 h and washed thrice with 0.01 M PBS. The samples were dehydrated with 30, 50, 70, 80, 90, 95, and 100% water–ethanol solutions for 10 min, respectively, and replaced twice with isoamyl acetate for 20 min each. After natural air drying in a superclean bench, the dry specimens were fixed on a support, coated with gold, and then observed.

The suspensions were fixed and dehydrated as described above. At 37 °C, the samples were treated with a 1:1 (*V*/*V*) acetone and embedding agent mixture for 2 h; then, they were treated with the 1:2 (*V*/*V*) aforementioned mixture overnight. After the samples were treated with the pure embedding agent for 5 h, they were inserted into the embedding plate and incubated at 37 °C overnight. Then, the embedding plate was placed in a 60 °C oven to polymerize for 48 h, the polymerized samples were sliced by an ultra-thin slicer to obtain ultra-thin sections ranging from 60 to 80 nm, and a 150-mesh copper mesh was used to collect the slices. Then, they were stained with 2% uranium acetate saturated alcohol solution and 2.6% lead citrate solution for 8 min each. Finally, they were dried at room temperature and observed by TEM (HT7800, Hitachi Limited, Tokyo, Japan).

### 2.4. DNA and Protein Leakage

BGC bacterial cells were harvested by centrifugation at 5000× *g* for 10 min, and the absorbance of samples at 260 nm was assayed using NanoDrop (NanoDrop One, Thermo Scientific, Waltham, MA, USA) to measure the DNA concentration.

The protein content was measured by the Total Protein Assay Kit (Bicinchoninic Acid method) (Nanjing Jiancheng Bioengineering Institute, Lot: A045-3-1, Nanjing, China) according to the instructions. Then, the absorbance of the samples was assayed at 562 nm using a microplate reader (Spark, Tecan Austria GmbH, Groedig, Austria), and the protein content was calculated according to the standard curve.

### 2.5. ATP Content

BGC bacterial cells were harvested by centrifugation at 5000× *g*, for 10 min, and were resuspended in 500 μL ddH_2_O. The ATP content was assayed following the instructions of the ATP assay kit (Nanjing Jiancheng Bioengineering Institute, A095-1-1). Then, the absorbance at 636 nm was measured on a microplate reader (Spark, Tecan Austria GmbH, Groedig, Austria), and the ATP concentrations were calculated referring to the standard curve.

### 2.6. ROS Release

The ROS concentrations were monitored by the Reactive Oxygen Species Assay Kit (Nanjing Jiancheng Bioengineering Institute, Lot: E004-1-1) according to its product manual. BGC bacterial cells were harvested by centrifugation (5000× *g* and 10 min) and subjected to incubation with the 2′,7′-dichlorodihydrofluorescein diacetate (DCFH-DA) probe. After incubation, the mixtures were washed 3 times with PBS to remove the free probe. Then, the fluorescence intensity of the DCFH-DA probe in the samples was measured at an excitation wavelength of 488 nm and an emission wavelength of 525 nm. Samples that were untreated by PL were set as the control, and their ROS release was set as 1. The ROS release of the PL-treated sample relative to the control was calculated and expressed as a percentage.

### 2.7. Color and Texture Change

To explore the impact of PL treatment on the quality of AA and AC, sterile (121 °C for 10 min) and rehydrated pieces (in sterile water for 12 h) of *Auricularia* were subjected to PL treatment at 11.80~35.40 J/cm^2^ on Petri dishes (60 mm). Samples were directly photographed for observation of changes in their appearance before and after PL treatment.

The L*, a*, and b* values were also recorded by a high-quality spectrophotometer, NS800 (3 nh, Shenzhen, China), to evaluate color changes before and after PL treatment. A texture analyzer (TA.XT Plus C, Stable Micro Systems, London, UK) was used to analyze changes in the texture of *Auricularia*. The TPA program and a P2 probe were used. Specifically, the speed before and after penetration was 2 mm/s, the measured speed was 1 mm/s, the trigger force was 5 g, and the residence time was 2 s. The shape variables of AA and AC were 50% and 75%, respectively. The samples were cut with in consistent 1 cm × 1 cm pieces for determination.

### 2.8. Statistical Analysis

Differences in inactivation effect, DNA, and protein leakage, ATP content, ROS release, and quality change under different treatments were compared by one-way ANOVA (Graphpad Prism 10.1.2) and Tukey’s test, and different letters represent significant differences among the test results when *p* < 0.05.

## 3. Results and Discussion

### 3.1. Impact of Initial Microbial Biomass and PL Parameters on Inactivation Efficacy

**Figure 1:**
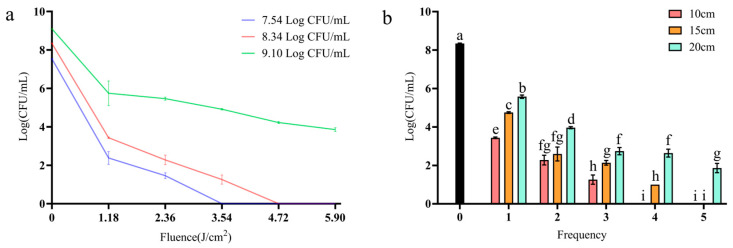
The impact of the initial microbial biomass (**a**) and number of flashes on the inactivation efficacy of pulsed light at differing flash distances (**b**).

It is shown in Figure 1a that higher BGC biomasses increased the difficulty of inactivation. The fluences needed for completely inactivating initial microbial biomasses of 8.34 ± 0.02 Log CFU/mL and 7.54 ± 0.02 Log CFU/mL were 4.72 J/cm^2^ and 3.54 J/cm^2^, respectively. However, inactivation was less effective at a 9.10 ± 0.01 Log CFU/mL initial microbial biomass, with 3.86 ± 0.10 Log CFU/mL remaining in the group treated with 5.90 J/cm^2^.

We evaluated the effect of different PL distances from the samples on the inactivation of BGC. Distances of 10, 15, and 20 cm were to be compared using a uniform initial microbial biomass of 8.34 ± 0.02 Log CFU/mL, as shown in Figure 1b. There were significant differences in the number of surviving bacteria at different flash distances (*p* < 0.05), except for the case of two flashes at distances of 10 cm and 15 cm. The reductions in bacteria count were 2.77~6.48 Log CFU/mL at 20 cm and 3.59~8.34 Log CFU/mL at 15 cm, and there was complete inactivation of the 8.34 initial biomass, with only four flashes needed (3.16 J/cm^2^). Shortening the flash distance within the effective range of PL can significantly enhance inactivation.

The effect of the number of flashes on the inactivation of BGC by PL is shown in Figure 1b. It can be clearly seen that, as the number of flashes increases, the number of surviving bacteria continuously decreases. There were significant differences in the number of surviving bacteria after different numbers of flashes (*p* < 0.05) at the same distance. The number of flashes required for complete inactivation of 8.34 ± 0.02 Log CFU/mL of bacteria increased from four to five (3.16 to 3.95 J/cm^2^) at a 15 cm distance.

Studies have shown that high spore biomass has no shielding effect on PL inactivation efficacy, which is comparable to the effect at low concentrations [23]; however, we obtained contradictory results as higher initial biomass reduces the inactivation efficiency. This may be because the structure and component of the rod-shaped BGC is less stress-bearing than spores. Because a large number of bacteria will cluster, when one flash is performed, the cells in the middle will survive, and these protected bacteria will be separated from the dead cells and be killed thereafter when more flashes are applied. The cumulative effect of the light flashes will enhance the bactericidal effect [24,25,26]. Under the same concentration and conditions, spherically shaped bacteria solutions may show a more uniform light-shielding effect due to higher particle density and isotropic scattering. However, the rod-shaped bacteria solution may have stronger shielding in a specific direction [27]. The actual differences may also be affected by the species specificity (such as flagella and capsule) and require further systematic experimental data. Studies have shown that, the closer the sample is to the light, the larger the fluence values and the larger the thermal component [28,29]. However, some studies have shown that distance has no significant effect on the number of surviving *Salmonella* [24]. It should be noted that the range of distance in their study was 30–90 mm, shorter than the minimal distance here. Biofilm, compared to vegetative cells, is a much more resistant to inactivation.

### 3.2. Inactivation of BGC on Auricularia

**Figure 2:**
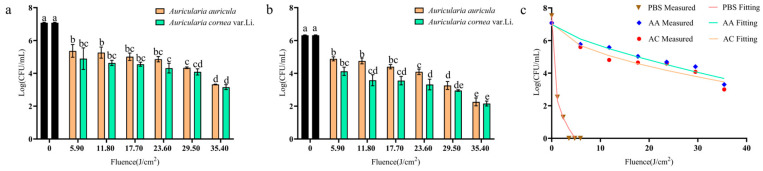
The inactivation of BGC on *Auricularia auricula* (AA) and *Auricularia cornea* var. Li. (AC) substrates under different initial microbial concentrations. (**a**) Initial microbial biomass at 7.08 ± 0.04 Log CFU/mL; (**b**) initial microbial biomass at 6.32 ± 0.02 Log CFU/mL; (**c**) fitting of Weibull model for three substrates.

The inactivation efficacy of PL treatment on BGC cells on the substrates AA and AC was investigated. An initial microbial biomass of 7.08 ± 0.04 Log CFU/mL and a flashing distance of 10 cm were taken; see Figure 2a. It was shown that, when the fluence increases from 5.9 to 35.4 J/cm^2^, the BGC biomass reduction in AA was from 1.72 ± 0.39 Log CFU/mL to 3.76 ± 0.04 Log CFU/mL. The reduction was better on AC, as 2.19 ± 0.66 Log to 3.92 ± 0.16 Log reductions were observed.

Then, the initial microbial biomass was reduced to 6.32 ± 0.02 Log CFU/mL while keeping the other parameters constant. Figure 2b shows that, when the fluence increased from 5.9 to 35.4 J/cm^2^, the BGC biomass reduction was from 1.44 ± 0.12 Log to 4.06 ± 0.24 Log on AA. Identically, slightly higher BGC reductions from 2.2 ± 0.25 Log to 4.16 ± 0.15 Log on AC were seen. Comparable biomass reduction results were also reported in *Salmonella* in pecan halves [14] and *Alicyclobacillus* in apple juice [30]. Further research on emerging non-thermal sterilization technologies targeting BGC is needed to evaluate and compare the advantages of PL methods.

Therefore, it can be seen that PL was effective in killing BGC on the *Auricularia* substrates. There was no significant difference in the bactericidal effect between the two kinds of substrates at a higher concentration of biomass (7 Logs) (*p* > 0.05); see Figure 2a. At a lower initial bacterial biomass (6 Logs) (Figure 2b), there was a significant difference in the bactericidal effect between the two substrates (*p* < 0.05) when the number of fluence was less than 17.70 J/cm^2^. The bactericidal effect on white ones (AC) was better than that on the black ones (AA).

### 3.3. Inactivation Model Fitting

The log-linear and Weibull models stood out among the 10 models available in GInaFiT, showing a better fit on the survivor curves. Weibull was the best-fitting model for the three substrates. The RMSE was 0.308~0.430, and the R^2^ values were between 0.943 and 0.987 (Table 1). On the whole, the fitted curves present a concave upward shape, and the curve is relatively gentle for AA and AC (Figure 2c). The results indicated that sensitive individuals among the BGC died out, while the remaining individuals increased their resistance to PL [31].

For different substrates, the shapes of the curve are different. BGC was more easily inactivated in PBS. Only 0.02 J/cm^2^ (*δ*) of fluence was needed to inactivate the bacteria in the first log cycle. The lowest *p* value (0.257 ± 0.058) of the model corresponded to a large arc in the concave curve, indicating that, when the fluence began to rise, a large number of bacteria died quickly, leading to a significant increase in the steepness of the curve’s front. Once the fluence was > 3, all the bacteria were killed, and no trailing was observed. A reason may be that PBS substrate does not have particles that block PL, making it easy for light radiation to penetrate. However, the larger *δ* values (3.379 ± 2.456~6.853 ± 3.110 J/cm^2^) obtained on *Auricularia* substrates was an indication of the difficulty of PL application on solid surfaces. The remaining surviving bacteria on AA and AC were both about 3 Logs. It is suggested that the probable mechanism of bacterial resistance to light may be the photoprotective properties of secondary metabolites [32,33] and the photoreactivation mechanisms that repair DNA [34]. In addition, variations in cell wall composition and structure [35] as well as clustering ability (shielding effect) contribute to this. The most likely reason for this study is due to the shielding effect of the *Auricularia* substrate. The fungus itself is very irregular in shape, leaving shadow areas. In addition, AA has a large amount of melanin [36], which consists of indole polymers, giving it a black color and imparting antioxidative activity and protection against radiation [37]. The absorption of light by AA may be more significant, which can reduce the radiation intensity of PL. In addition, the surfaces of AA and AC differ in that AA is more wrinkled, while AC is smooth. This is also confirmed by the better bactericidal effect of the white colored ones (AC) than black ones (AA). Therefore, the physical form of food substrate has great influence on the PL’s effect. This is in accordance with the conclusion of Hee-Jeong Hwang that the roughness of food surfaces impacts germicidal efficacy [38]. It was further proven that rough and uneven stem scars on tomatoes shielded *Salmonella enterica* [39]. Moreover, the implication that PL-induced damage in bacteria is reduced when located in PL-absorbing food substrates that are rich in protein, fat, and water [24,40] is also possible. Thus, these factors may be the reasons for the ineffective inactivation of the remaining bacterial cells. Besides the chemical component variation in the two *Auricularia* species, their physical thickness also differs, with AC generally being thicker than AA. The exact reasons for this disparity in the bactericidal effect on the two species still needs further investigation with a focus on the abovementioned factors.

### 3.4. Impact of PL on Bacterial Cell Morphology

**Figure 3:**
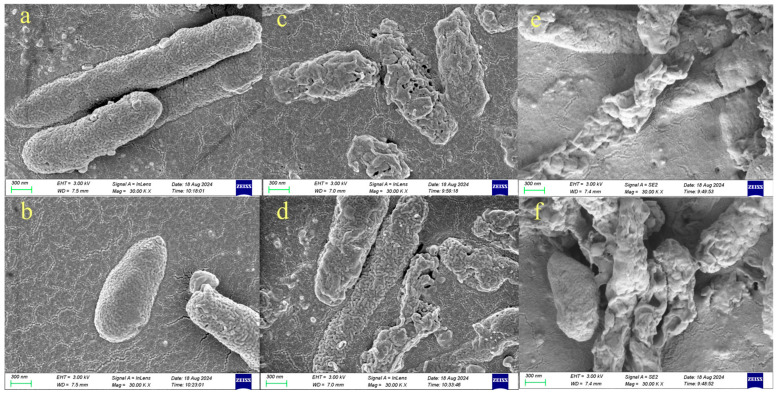
Cell morphology of BGC by SEM under pulsed-light treatment ((**a**,**b**) untreated BGC cell morphology; (**c**,**d**) BGC cell morphology after 2.36 J/cm^2^ treatment; (**e**,**f**) BGC cell morphology after 5.90 J/cm^2^ treatment).

The bacterial cell morphology of BGC was observed by SEM before and after PL treatment at 2.36 and 5.90 J/cm^2^ fluences. As can be seen from Figure 3a,b, untreated BGC presented a rod-shaped and intact appearance. The cell surfaces were round and smooth, and the membranes were continuous. However, the bacterial cell surface showed wrinkles, collapse, and shrinkage when pulsed-light treatment was applied (Figure 3c,d). The integrity of the membranes was compromised in some cases. When more fluences were applied, the bacterial cells collapsed further, and cytoplasmic shrinkage occurred (Figure 3e,f). The structure and the morphology of the bacterial cells were heavily damaged, and the cellular content was lost.

**Figure 4:**
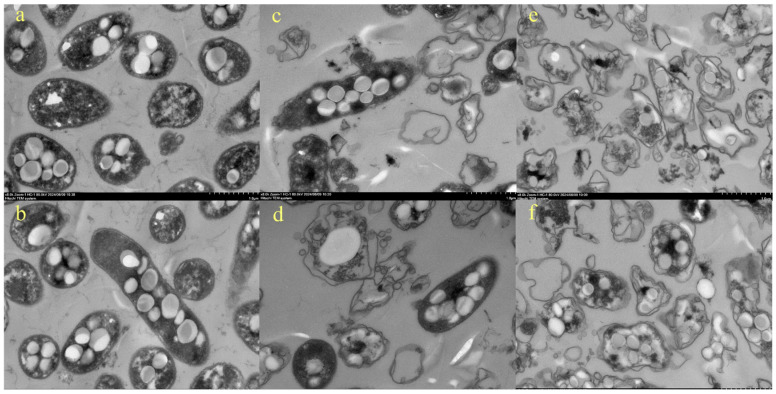
Cell morphology of BGC by TEM under pulsed-light treatment ((**a**,**b**) untreated BGC cell structure; (**c**,**d**) BGC cell structure after 2.36 J/cm^2^ treatment; (**e**,**f**) BGC cell structure after 5.90 J/cm^2^ treatment).

The cell structures were also observed by TEM. The untreated BGC cells showed structural integrity. The structures of the plasma membranes were intact. Distinct cell wall and membrane bilayer structures were seen from the cells (Figure 4a,b). Transparent and translucent polyhydroxyalkanoates granules were also evident. The genus *Burkholderia* has been reported to accumulate storage polyesters when growth is constrained by the absence of an essential nutrient and the carbon source is sufficient [41]. Cytoplasm was also observed in the cells. However, the cell membrane of the bacteria was broken, and the contents of the cells began to leak after 2.36 J/cm^2^ treatment (Figure 4c,d). This worsened when 5.90 J/cm^2^ was applied (Figure 4e,f), where bacterial cells were severely damaged. A large amount of cytoplasmic component leakage was observed, and the cells were surrounded by them. The changes in cell structure were consistent with the cell morphology in SEM pictures. They all proved that bacterial cells were damaged by PL and led to subsequent cell death.

Our SEM and TEM results agree that PL destroyed the integrity of bacterial cells. Contradictorily, researchers ascribed the lack of obvious cellular damage to the low detection sensitivity of TEM due to prolonged sample preparation [42]. Our SEM results provide clarification, proving that the cell rupture was due to the action of PL rather than the sample preparation process. The results support that photophysical effects play a primary role in inactivation.

### 3.5. DNA and Protein Leakage, ATP Content, and ROS Release

**Figure 5:**
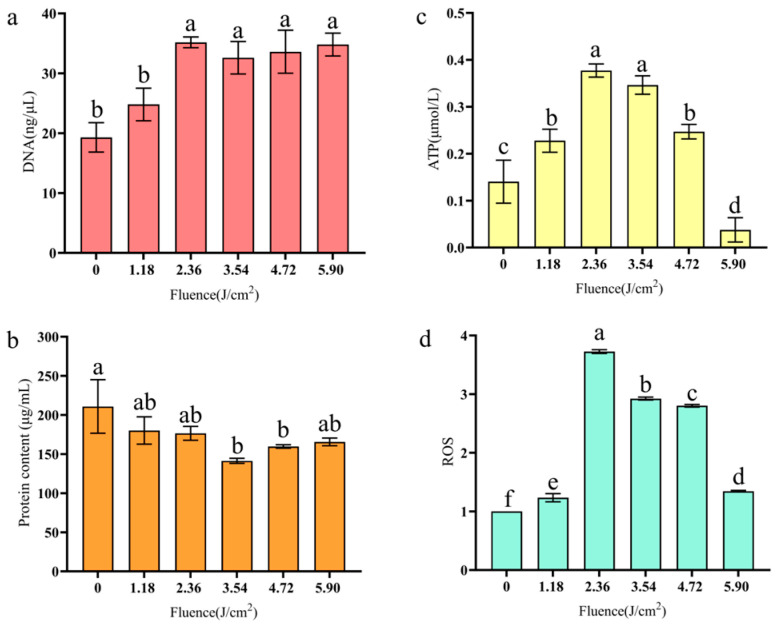
Changes in DNA leakage (**a**), protein leakage (**b**), ATP content (**c**), and ROS release (**d**).

The effects of PL on the DNA and protein contents of BGC are shown in Figure 5. Figure 5a clearly shows DNA leakage from BGC after PL treatment. Compared with the untreated group (0 J/cm^2^), the DNA content of BGC was significantly increased after >2.36 J/cm^2^ treatment. There was no significant change in DNA content as the fluences increased from 2.36 to 5.9 J/cm^2^, and at 5.9 J/cm^2^, the DNA content was increased by 15.5 ng/μL (*p* < 0.05). This showed that PL treatment could destroy the structure of the cell membrane, leading to the leakage of intracellular content and having an adverse effect on cell survival.

Figure 5b shows the protein content of BGC cells after being treated with PL. The amount of intracellular protein leakage and the degree of cell rupture increased with the fluence applied within the range of 0~2.36 J/cm^2^. It was inferred that the rate of protein destruction by PL was greater than the rate of intracellular protein leakage; indeed, the overall protein content showed a decreasing trend (*p* < 0.05).

The change in ATP content in BGC treated by PL is shown in Figure 5c. The ATP content of BGC was firstly increased from 0 to 2.36 J/cm^2^, and then, it was decreased from 3.54 to 5.90 J/cm^2^. After PL treatment at 2.36 J/cm^2^, the ATP content increased by 0.237 μmol/L. When the fluence reached 5.90 J/cm^2^, the ATP content was decreased by 0.103 μmol/L compared with the control group (*p* < 0.05). The change in ATP content during the treatment process may be because PL stimulated the mitochondrial electron transport chain in BGC cells, thereby increasing ATP production and contributing to the formation of intracellular ROS. However, with the increase in PL intensity, the cells gradually entered apoptosis, and the ATP content was also decreased.

As can be seen in Figure 5d, at the beginning of PL treatment, the total amount of ROS in BGC cells increased significantly with the increase in intensity. At 2.36 J/cm^2^, the ROS level rose to 3.75 times that of the control group, due to the fact that ultraviolet light can induce the formation of ROS in cells. ROS react with various cell components, resulting in lipid peroxidation damage and destruction of structural proteins, enzymes, and nucleic acids [43]. At 3.54 J/cm^2^, ROS levels began to decline, possibly due to the presence of excess ROS causing a change in cell permeability and cell apoptosis, and intracellular ROS were released (*p* < 0.05).

BGC is a Gram-negative bacterium. Studies have shown that Gram-negative bacteria are more sensitive to PL than Gram-positive bacteria, because Gram-negative bacteria do not have the thick peptidoglycan layer present in positive bacteria, which may reduce the absorption of PL by cells and DNA [42,44].

It has been reported that the main effect of PL is a photochemical reaction as the maximum absorbance of DNA occurs in the 260–265 nm range [15]; the damage on these macromolecules hinders the occurrence of a photoreactivation reaction. Our results support a photophysical effect more than they do a photochemical effect as the increase in free DNA after PL may be due to the outflow of intracellular DNA accompanied by structural disintegration. In addition, ROS production may affect respiratory enzyme activity and membrane function, counteracting DNA repair [45]. However, we did not observe this phenomenon, which further confirms that the bactericidal mechanism was mainly based on physical destruction. The protein content was decreased slightly after treatment, and it was decreased significantly at 3.54 and 4.72 J/cm^2^. A possible cause is the breakdown of cellular structure, and Zhu has obtained similar results [46]. Noriyuki also found that ATP would increase and then decrease after cell death [47]. Due to the destruction of the integrity of the cell membrane, the ion gradient inside and outside the cell disappears, the release and activation of intracellular ATPase occur, and some metabolic pathways such as glycolysis still make use of surplus glucose to generate ATP. With the addition of the release of the ATP pool, all the factors will cause the ATP level to rise briefly and drop rapidly thereafter [48], an identical observation to the results here.

### 3.6. Color and Texture Measurements

**Figure 6:**
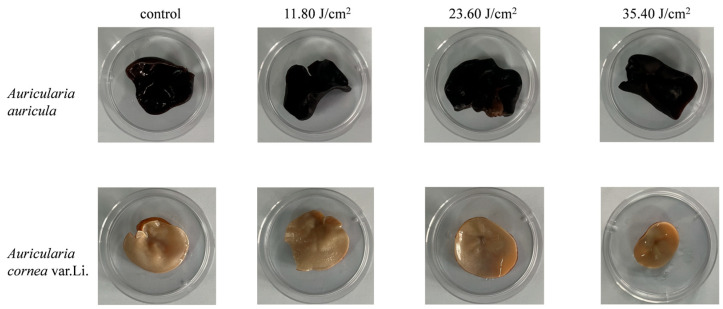
The appearance of *Auricularia auricula* (AA) and *Auricularia cornea* var. Li. (AC) before and after pulsed-light treatment.

We used a camera to record the color changes in *Auricularia* before and after PL treatment. As shown in Figure 6, no obvious changes can be seen with the naked eye across fluences ranging from 11.8 J/cm^2^ to 35.4 J/cm^2^. Table 2 shows the color changes recorded by a spectrophotometer. It was found that, for AA, the 11.8 J/cm^2^ treatment caused significant changes in color. These color changes were mainly due to increased luminosity (L*) and reduced a* and b* caused by PL processing. At a small fluence of 11.8 J/cm^2^, the PL treatment made the color of AA redder (a*) and yellower (b*). For the light-colored AC, the color change was significant under 11.8~35.4 J/cm^2^ PL treatment. The main reason for this change is the reduction in yellow coloration (b*). The color change decreased as increasing fluence was applied in the range of 11.9~35.4 J/cm^2^ fluence. At 35.40 J/cm^2^, the minimum ΔE was observed, where the inactivation efficacy was also optimal (Figure 2). The color change in AC after PL was greater than that in AA. The color change in the AC was apparent (3 < ΔE < 5), while AA only had a slight difference in color (2 < ΔE < 3). Therefore, the PL treatment led to varying degrees of color variation, though this is not easily detectable by the naked eye (Figure 6). Studies have also reported that PL could lead to noticeable color changes (ΔE 6.1 and 8.5) in wheat flour and black pepper at 31.12 J/cm^2^ comparable to but greater than those observed in this study [49]. In brief, these color changes may be due to the thermal effects of PL, so the necessary cooling devices need to be added.

**Figure 7:**
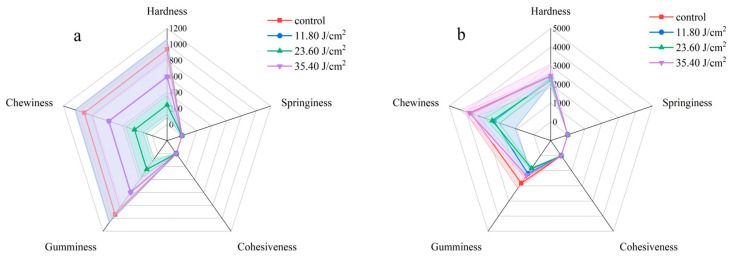
Texture changes in *Auricularia auricula* (AA) (**a**) and *Auricularia cornea* var. Li. (AC) (**b**) before and after pulsed-light treatment.

Figure 7 shows the texture changes in *Auricularia* before and after PL treatment. We measured hardness, springiness, cohesiveness, gumminess, and chewiness for a comprehensive description of the texture. There was a big difference between the two *Auricularia* varieties. AC was harder (hardness), more springy, more gummy, and more chewy than AA. PL treatment with varying fluences (0 or 11.8~35.4 J/cm^2^) did not significantly change the cohesiveness of AA and AC (*p* > 0.05). Cohesiveness is a parameter describing how food holds together or the difficulty in breaking its structure [50]. The springiness of AA was also not influenced by PL treatment, and this was also the case for AC. Similarly, there was no significant difference in the hardness in AA and AC before and after PL treatment. It should be noted that the large variation in replicate samples produced this insignificance in AA. This also happened to gumminess and chewiness for both varieties.

PL was found to affect the texture of fresh-cut mushrooms (*Agaricus bisporus*) due to thermal damage [51]. Research has also reported the deterioration of texture by PL in yellow croakers during cold storage [52]. Consumers prefer the crunchy and smooth or tough texture of *Auricularia*, which was partially maintained and reflected in the unchanged springiness and cohesiveness. Because of the irregular shape of the fruit body of the fungus, individual differences in *Auricularia* and its varieties cannot be ignored. Generally speaking, PL treatment maintained the springiness and cohesiveness of *Auricularia*.

## 4. Conclusions

This study investigated the feasibility of using PL for the inactivation of BGC, and ~4 Log reductions in *Auricularia* were observed. It was inferred that the bactericidal mechanism was mainly based on photophysical effects. PL led to rupture in bacterial cells, the outflow of internal biomacromolecules, and ROS production. PL led to color change (ΔE 3.01~7.67) but not changes in the texture of *Auricularia*. A PL fluence of 35.4 J/cm^2^ was suggested for the inactivation of BGC in *Auricularia*. In the future, combinations of other sterilization methods can be considered to reduce the adverse impact on the color of this food.

## Figures and Tables

**Table 1:** Values of model parameters obtained by fitting the survivor curves from PL treatment in PBS, *Auricularia auricula* (AA), and *Auricularia cornea* var. Li. (AC) to different kinetic models.

Substrate	Parameters	Model					
		Log-Linear	RMSE	R^2^	Weibull	RMSE	R^2^
PBS	*k_max_* [cm^2^/J]	2.592 ± 0.817	1.752	0.715	-	0.430	0.987
	Log_10_*N*_0_ [CFU/mL]	5.212 ± 1.268			7.530 ± 0.430		
	*δ* [J/cm^2^]	-			0.002 ± 0.003		
	*p* [-]	-			0.257 ± 0.058		
*Auricularia auricula*	*k_max_* [cm^2^/J]	0.209 ± 0.023	0.314	0.942	-	0.308	0.955
	Log_10_*N*_0_ [CFU/mL]	6.724 ± 0.214			6.986 ± 0.302		
	*δ* [J/cm^2^]	-			6.853 ± 3.110		
	*p* [-]	-			0.730 ± 0.179		
*Auricularia cornea* var. Li.	*k_max_* [cm^2^/J]	0.216 ± 0.034	0.464	0.888	-	0.372	0.943
	Log_10_*N*_0_ [CFU/mL]	6.491 ± 0.316			7.040 ± 0.369		
	*δ* [J/cm^2^]	-			3.379 ± 2.456		
	*p* [-]	-			0.543 ± 0.154		

*N*_0_, initial biomass (CFU/mL); *k_max_*, specific inactivation constant of the model (cm^2^/J); *p*, shape parameter of the model; *δ*, fluence of the first log cycle reduction; RMSE, root mean square error; R^2^, correlation coefficient.

**Table 2:** Values of colorimeter parameters obtained by *Auricularia auricula* (AA) and *Auricularia cornea* var. Li. (AC).

Substrates	Indicators	Treatment			
		Control	11.80 J/cm^2^	23.60 J/cm^2^	35.40 J/cm^2^
*Auricularia auricula* (AA)	L*	17.03 ± 0.55 ^b^	20.81 ± 0.38 ^a^	21.37 ± 1.56 ^a^	20.22 ± 0.87 ^a^
	a*	3.35 ± 0.26 ^a^	1.35 ± 0.27 ^b^	2.43 ± 0.31 ^a^	2.81 ± 0.28 ^a^
	b*	2.87 ± 0.29 ^a^	1.14 ± 0.38 ^b^	2.03 ± 0.09 ^ab^	2.68 ± 0.49 ^a^
	ΔE	0.41 ± 0.63 ^b^	4.50 ± 0.47 ^a^	4.34 ± 1.34 ^ab^	3.01 ± 0.93 ^ab^
*Auricularia cornea* var. Li. (AC)	L*	56.49 ± 0.70 ^a^	49.67 ± 0.53 ^b^	58.93 ± 1.06 ^a^	55.60 ± 0.31 ^a^
	a*	1.07 ± 0.10 ^b^	2.86 ± 0.30 ^a^	1.92 ± 0.10 ^a^	0.65 ± 0.27 ^c^
	b*	12.25 ± 0.62 ^a^	8.19 ± 0.50 ^b^	8.24 ± 0.80 ^b^	8.53 ± 0.13 ^b^
	ΔE	0.80 ± 0.74 ^c^	7.67 ± 0.23 ^a^	5.21 ± 1.26 ^ab^	3.89 ± 0.17 ^b^

## Data Availability

Data will be made available on request.
